# Hepatitis B and C: Seroprevalence, knowledge, practice and associated factors among medicine and health science students in Northeast Ethiopia

**DOI:** 10.1371/journal.pone.0196539

**Published:** 2018-05-15

**Authors:** Wondmagegn Demsiss, Abdurahaman Seid, Temesgen Fiseha

**Affiliations:** Department of Medical Laboratory Sciences, College of Medicine and Health Sciences, Wollo University, Dessie, Ethiopia; Centre de Recherche en Cancerologie de Lyon, FRANCE

## Abstract

**Background:**

Health care professionals, especially medical students, are at greater risk of contracting hepatitis B and C virus infections due to their occupational exposure to percutaneous injuries and other body fluids. The aim of this study was to determine the seroprevalence of hepatitis B and C virus infections among medicine and health science students in Northeast Ethiopia and to assess their knowledge and practice towards the occupational risk of viral hepatitis.

**Methods:**

A cross-sectional study was conducted among a total of 408 medicine and health science students during the period from March to September 2017. A pre-coded self-administered questionnaire was used to collect data on students’ socio- demographic characteristics, knowledge and practice of hepatitis B and C infections. Blood samples were collected and screened for hepatitis B surface antigen (HBsAg) and anti-HCV antibodies. SPSS version 20 statistical software was used for data analysis.

**Results:**

The seroprevalence of HBV infection was 4.2% (95% CI 2.5 to 6.1%) and 0.7% (95% CI 0.0 to 1.7%) for HCV. Older age (AOR = 15.72, 95% CI 1.57–157.3) and exposure to needlestick injury (AOR = 3.43, 95% CI 1.10–10.73) were associated with a higher risk of HBV infection. Majority of the students (80.1%) had an adequate knowledge about hepatitis B and C infection, mode of transmission and preventive measures. Only 50.0% of students had safe practice towards occupational risk of viral hepatitis infection. Almost half (49.8%) of students experienced a needlestick injury; of which, 53.2% reported the incidence, and only 39.4% had screening test result for viral hepatitis.

**Conclusion:**

A high seroprevalence but poor practice of hepatitis B and C virus infection was found in the study area despite their good knowledge towards occupational risk of viral hepatitis infection.

## Introduction

Viral hepatitis due to hepatitis B and C is a global public health problem affecting millions of people worldwide, causing an estimated 1.3 million deaths each year from acute infection and hepatitis-related liver cancer and cirrhosis [1]. It is estimated that more than two billion people are infected with hepatitis B virus (HBV) worldwide and about 350 million of them suffer from chronic HBV infection [2]. There are also about 177.5 million carriers of hepatitis C virus (HCV), resulting in about 350,000 global deaths annually [3,4]. The burden of hepatitis B and C infection is highest in the developing world and mostly affects resources limited countries, where screening and access to care and treatment are not readily available [5]. The burden of viral hepatitis in Ethiopia is highest; where an estimated 6.5–8.4% of the population is infected with HBV and 2.2–4.4% with HCV[6].

Viral hepatitis B and C are blood-borne infections, with significant transmission occurring through unsafe injections and medical procedures, and less commonly through sexual contact. Health-care workers are, thus, at greater risk of acquiring these two blood-borne infections from percutaneous (needlestick/sharp instrument) injuries or other types of occupational exposures, and the incidence of this infection among them has been estimated to be four times the level in the general population [7,8]. Needlestick accidents with percutaneous inoculation is a well-documented HBV and HCV transmission, with seroconversion rate of 10–30% following needle stick exposure to HBV infected blood and about 2.0% for HCV [9]. Overall the number of health-care workers annually exposed to sharp injuries contaminated with HBV and HCV was estimated to reach 2.1 million and 926,000, respectively [10]. Protection of health care workers through immunization (for HBV), use of protective equipment and post-exposure management is therefore critical to prevent occupational risk of exposure to and transmission of these blood-borne viruses [11].

Medical students are a group of health care workers that are at high risk to get HBV and HCV infections because of their direct contact with patients, blood and other body fluids during their professional training, and lack of experience and professional skills increases the risk of infection in the course of invasive medical procedures [12]. Medical students receive percutanous injuries as often or more than health care workers and are, therefore, at greater risk of occupational exposure to HBV and HCV infections than health care workers, a fact that might partially be explained by poor knowledge and non-adherence to universal infection control procedures [13,14]. In addition to this, there exists a widely prevalent problem of underreporting of this risk exposures by students, which represents a missed opportunity for initiating post exposure prophylaxis, early detection of seroconversion and implementation of prevention strategies [15,16]. All these can lead to the risk of the medical students being exposed to HBV and HCV to a level perhaps somewhat higher than that of average health care workers.

Even though studies assessing the knowledge, attitudes and practice of HBV infection have been conducted among Ethiopian medical and health science students [17,18], there has been no study assessing the seroprevalence of HBV and HCV among health students in Ethiopia despite this high risk of exposures. Therefore, this study was carried out to determine the seroprevalence of hepatitis B and C virus infections among medicine and health science students in Northeast Ethiopia and to evaluate the knowledge and practice of students towards the occupational risk of blood-born viral hepatitis infection.

## Materials and methods

### Setting, inclusion and exclusion criteria

This cross-sectional study was conducted at the College of Medicine and Health Sciences, Wollo University during the period from March to September 2017. The university is located in South Wollo Zone of the Amhara regional state, Northeast Ethiopia. College of medicine and health sciences is the largest college in the university and one of medical education center in the country. It is located in Dessie campus 4 kilometer away from Dessie town and 400 kilometers away from the capital Addis Ababa.

All voluntary Medicine and Health Science students of Wollo University who have started clinical practice were included in this study. Those students who were not on clinical attachment, those who have mental illness, seriously ill and unable to give consent were excluded from the study.

### Sample size determination

A single population proportion formula was used to estimate sample size. Since there was no similar study from Ethiopia, the following assumptions have been made: 95% confidence interval (Z_α/2_ = 1.96), 50% proportion (P), and 5% margin of error (d).

$$n=\frac{{\left(Z_{\alpha /2}\right)}^{2}\times P\left(1-P\right)}{d^{2}}$$

$$n=\frac{{\left(1.96\right)}^{2}\times 0.5\left(1-0.5\right)}{0.05^{2}}=384$$

Where n = sample size. By adding 10% for non-response rate, the estimated sample size was 422 study participants. The sample size was proportionally distributed to each department. The study participants were selected by a random sampling technique considering the characteristics we are going to study is uniformly distributed among the study population.

### Study variables

Hepatitis B and C sero-positivity, knowledge and practice of the study participants towards transmission and prevention were considered as dependent variables, and age, sex, residential area, marital status, previous work area/practical place, year of study and departments of the study population were considered as the independent variables.

### Data collection process

A self-administered structured questionnaire was used to collect information about the socio-demographic characteristics (age, sex, marital status, residence, and practical/work place), knowledge like cause, transmission and prevention methods of hepatitis B and C viruses and practice towards prevention of these two blood-borne viral infections. The English version of the questionnaire was used to collect the information from the respondents. The pre-designed questionnaire was pre-tested on 5% of clinical students in the college other than the study participants and necessary modifications were made.

### Definitions for scoring knowledge and practice

The following operational definitions for scoring knowledge and practices of students were used in this study. Good knowledge: if the respondents were able to answer 70% or more of knowledge items correctly. Poor knowledge: if the respondents answered less than 70% of knowledge items. Good practice: when the study participants were at least able to answer 70% or more practice items on the questionnaire correctly. Malpractice: when the participants were unable to answer 70% of practice items correctly [17].

### Laboratory investigations

Five ml of venous blood samples were collected into plain tubes from each study subjects and were allowed to clot at room temperature for 30 minutes. Samples were then centrifuged at 10,000 rpm for 10 min, and serum was separated for testing HBV and HCV. Sero-positivity for HBV infection was detected using the LINEAR HBsAg Cassette test (Linear Chemicals SL, Barcelona, Spain) and was performed and interpreted in accordance with the manufacturer’s instruction. This is a rapid one step membrane-based immunodiagnostic assay designed for qualitative determination of hepatitis B surface antigen (HBsAg) in human serum. HCV cassette antibody test (Linear Chemicals SL, Barcelona, Spain) was used to detect positivity for HCV infection. As part of the quality control we utilized known positive and negative controls from patient sample verified by ELISA.

### Statistical analysis

Data were entered and cleaned using “EpiData 3.1” software and analyzed using SPSS version 20.0 (SPSS, Chicago, IL, USA). Descriptive statistics like frequencies and proportions were used to summarize the data. Bivariate and multivariate analyses were used to examine the relationship between the dependent variables and selected socio-demographic factors. Adjusted odds ratios (AOR) and their 95% confidence intervals (CIs) were used as indicators of the strength of association. A P value of 0.05 or less was used to indicate statistical significance.

### Ethical consideration

The study was ethically approved by college of Medicine and Health Sciences Research Ethics review Committee at Wollo University. Written informed consent was obtained from each study participants. Moreover, confidentiality was assured for all the information provided and personal identifiers were not included on questionnaire. Those students who were positive were linked to clinicians in the student’s clinic for health education, counseling and other follow-ups.

## Results

### Socio-demographic characteristics

A total of 422 randomly selected students were invited to participate in the study of which 8 returned incomplete questionnaire and 6 were unwilling to give blood sample for laboratory investigation. Therefore 408 study subjects (96.7% response rate) with completed questionnaire and having laboratory investigation result were included in the final analysis. From the total study subjects 264 were male and 144 were female. The mean age of the students was 26.3 ± 3.7 years (ranging from 19 to 40 years), and 60.8% were between the age group of 25 and 34 years. Majority, (64%) of the respondents were urban residents. More than half of the students (52.7%) were BSc. nursing students. Medical laboratory, medicine, midwifery and pharmacy students were 22.8%, 10.3%, 8.8% and 5.4%, respectively. Majority of them (80.4%) were 3^rd^ year students and the rest 14.2%, 2.5% and 2.9% were 2^nd^, 4^th^ and 5^th^ year students, respectively. Of the total participants, 240 (58.8%) used to work or practice in primary health care centers, and 130(31.9%) and 38 (9.3%) worked in hospitals and private clinics/ laboratories, respectively in previous times. In relation to marital status, 229 (56.1%) of the participants were single, while 179 (43.9%) were married (Table 1).

**Table 1:** Socio-demographic characteristics of study participants at college of medicine and health sciences, Wollo University, Northeast Ethiopia, 2017. (n = 408).

Characteristics	Category	N (%)
**Age**, year, mean (SD)		26.3 ± 3.7
**Age group**	<25 Years	132 (32.4)
	25–34 Years	148 (60.8)
	≥35 Years	28 (6.9)
**Gender**	Male	264 (64.7)
	Female	144 (35.3)
**Residence**	Urban	261 (64)
	Rural	147 (36)
**Marital Status**	Married	179 (43.9)
	Single	229 (56.1)
**Place of work**	Hospital	130(31.9)
	Health center	240 (58.8)
	Private clinic/ laboratories	38 (9.3)
**Department**	Medical laboratory	93 (22.8)
	Medicine	42 (10.3)
	Midwifery	36 (8.8)
	Nursing	215 (52.7)
	Pharmacy	22 (5.4)
**Year of study**	2^nd^ year	58 (14.2)
	3^rd^ year	328 (80.4)
	4^th^ year	10 (2.5)
	5^th^ year	12 (2.9)

### Knowledge towards hepatitis B & C transmission and prevention

As indicated in Table 2 overall,331 (81.1%) of the study participants had adequate knowledge on hepatitis B & C infection, its mode of transmission and preventive measures. The mean knowledge score of the study participants was 13.4(SD+1.6).

**Table 2:** Correct response of the study participants to knowledge items towards hepatitis B and C at college of medicine and health sciences, Wollo University, Northeast Ethiopia, 2017. (n = 408).

Knowledge	No. (%)
Hepatitis can be caused by viruses	397 (97.3)
Hepatitis B & C can be transmitted through blood and blood products	398 (97.5)
Hepatitis B & C can be transmitted through needlestick or sharp object injury	395 (96.8)
Hepatitis B & C can be transmitted through sexual intercourses	323 (79.2)
Hepatitis B & C can be transmitted through feco-oral route	287 (70.3)
Hepatitis B & C can be transmitted from mother to child during pregnancy	346 (84.8)
Hepatitis B & C can be transmitted through close personal contact	164 (40.2)
Health student can acquire hepatitis B & C owning to their professional contact with patients-	332 (81.4)
A person can be infected and remain asymptomatic	225 (55.1)
Aware of the existence of a vaccine to prevent hepatitis B infection	342(83.8)
Hepatitis C infection can be prevented by vaccination	242(59.3)
Hepatitis B infection can be prevented by vaccination	351 (86.0)
Hepatitis B & C can be prevented by proper disposal of needle/ sharps	388 (95.1)
Hepatitis B & C can be prevented by avoiding casual sex or multi sexual partnership	355 (87.0)
There is treatment/ PEP for HBV	147 (36.0)
Hepatitis B & C can cause liver cancer	377 (92.4)
Good knowledge	331 (81.1)

Most of the students 397 (97.3%) knew that hepatitis B & C is caused by virus. The majority of the students 377 (92.4%) knew that HB/CV can cause liver cancer. Most of students correctly identified blood and blood products (97.5%, n = 398), injury with contaminated needles and sharps (96.8%, n = 395), sexual intercourse (79.2%, n = 323) and mother-to-child transmission (84.8%, n = 346) as routes of Hepatitis B and C virus transmission. Majority of the students (70.3%, n = 287) knew that Hepatitis B and C virus cannot be transmitted by fecoral and contaminated water. However, most of the students 59.8% (n = 244) incorrectly identified that hepatitis B and C can be transmitted through close personal contact like taking or kissing to an infected person, and 65.9% (n = 269) that hepatitis (B and C) is a nosocomial infection.

The majority 81.4% (n = 332) of them were aware of that health students can acquire hepatitis B and C owning to their professional contact with patients. However, only 63% (n = 257) of them had a positive attitude towards routinely testing all health students for HBV and HCV infection and restricting hepatitis B and C-positive students to low-risk procedures (47.1%, n = 192). Majority (83.8%, n = 342) of students were aware of the existence of a vaccine to prevent HBV infection and up to 86% (n = 351) knew that HBV infection can be prevented by vaccination. Majority of the study participants knew that hepatitis B and C can be prevented by proper disposal of needle/ sharps (95.1%, n = 388), avoiding needle/ sharps injury (94.6%, n = 386) and avoiding casual sex or multi sexual partnership (87%, n = 355).

Overall,331 (81.1%) of the study participants had adequate knowledge on hepatitis B & C infection, its mode of transmission and preventive measures (See Table 2).

### Practical measures for hepatitis B & C prevention and post-exposure behaviors

Of the 408 participants, 273 (66.9%) always change glove for each patient during blood collection and only 12 (2.9%) of the students did not use any gloves while handling different body fluids. 244 (59.8%) of the study participants properly dispose needle/ sharps, of which 150 (61.5%) are found in the age group of 25–34 followed by 79(32.4%) and 15 (6.1%) in the age group of <25 and ≥ 35 respectively. About 203(49.8%) of the students had history of needlestick injury. In the presence of this high level of exposure from students who had history of needlestick injury, only 110 (54.2%) knew that a person can be infected and remain asymptomatic, 108 (53.2%) reported the incidence, and only 80 (39.4%) had screening test results for HB/CV infection. Overall, there were unsatisfactory practical measures on prevention of hepatitis B and C infection among the study subjects (Table 3).

**Table 3:** Correct preventive of medicine and health sciences students towards hepatitis B and C at Wollo University, Northeast Ethiopia, 2017. (N = 408).

HB/CV practice questions	No. (%)
Always use glove while handling different body fluids	273 (66.9)
Always properly dispose needle/ sharps	244 (59.8)
Had a history of needlestick injury	203 (49.8)
Always report for needlestick injury	108 (53.2)
Have you ever screened for hepatitis B or C?	193 (47.3)
Overall good practice	204(50.0)

### Association of knowledge and practice with demographic characteristics

The univariate analysis showed that student’s residence and department were significantly associated with good level of knowledge towards transmission and prevention of hepatitis B and C infection (Table 4). However, none of these sociodemographic characteristics were significantly associated with knowledge of hepatitis B and C transmission and prevention in multivariate analysis. Among the demographic characteristics residence, department and place of work of the respondents were significantly associated with good practical measures towards hepatitis B and C prevention on univariate analysis. Multivariate analysis showed that residence of students was significantly associated with good practice towards prevention of hepatitis B and C infection (P = 0.001). Being urban resident is significantly associated with good practice towards prevention of hepatitis B and C infection as compared to rural resident (AOR 2.21, 95% CI 1.35–3.61).

**Table 4:** Analysis of sociodemographic characteristics with knowledge and practice, at college of medicine and health sciences, Wollo University, Northeast Ethiopia, 2017. (n = 408).

Variables		Knowledge			Practice		
		Good No (%)	Poor No (%)	P-value	Good No (%)	Poor No (%)	P-value
**Age group**	<25 Years	101(76.5)	31(24.9)	0.345	103(78)	29(22.0)	0.076
	25–34 Years	203(81.9)	45(18.1)		196(79.0)	52(21.0)	
	≥35 Years	27(96.4)	1(3.6)		27(96.4)	1(3.6)	
**Gender**	Male	220(83.3)	44(16.7)	0.123	204(77.3)	60(22.7)	0.073
	Female	111(77.1)	33(22.9)		122(84.7)	22(15.3)	
**Residence**	Urban	204(78.2)	57(21.8)	**0.041**	221(84.7)	40(15.3)	**0.001**
	Rural	127(86.4)	20(13.6)		105(71.4)	42(28.6)	
**Marital Status**	Married	142(79.3)	37(20.7)	0.412	147(82.1)	32(17.9)	0.322
	Single	189(82.5)	40(17.5)		179(78.2)	50(21.8)	
**Place of work**	Health center	192(80.0)	48(20.0)	0.379	209(87.1)	31(12.9)	**<0.001**
	Hospital	105(80.8)	25(19.2)		97(74.6)	33(25.4)	
	Private clinic	34(89.5)	4(10.5)		20(52.6)	18(47.4)	
**Department**	Medical Lab.	87(93.5)	6(6.5)	**<0.001**	75(80.6)	18(19.4)	**0.013**
	Medicine	34(81.0)	8(19.0)		30(71.4)	12(28.6)	
	Midwifery	35(91.7)	3(8.3)		31(86.1)	5(13.9)	
	Nursing	157(73.0)	58(27.0)		178(82.8)	37(17.2)	
	Pharmacy	20(90.9)	2(9.1)		12(54.5)	10(45.5)	
**Year of study**	2^nd^ year	50(86.2)	8(13.8)	0.411	41(70.7)	17(29.3)	0.109
	3^rd^ year	261(79.6)	67(20.4)		266(81.8)	62(18.9)	
	4^th^ year	9(96.0)	1(10.0)		10(100.0)	0(0.0)	
	5^th^ year	11(91.7)	1(8.3)		9(75.0)	3(25.0)	

### Seroprevalence of hepatitis B and C

From the total students participating in the study, seventeen (4.2%; 95% CI 2.5 to 6.1%) were positive for hepatitis B surface antigen (HBsAg) and three (0.7%; 95% CI 0 to 1.7%) for anti-hepatitis C (anti-HCV). The proportion of HBsAg positive cases was significantly higher (10.7%) in the older age groups of ≥35 years when compared with the other age groups; 5.2% for 25–35 years and 1.5% for <25 years (*P* = 0.023). However, there is significant difference in the proportion of HBsAg positive cases between males and females: 5.7% vs. 1.4% (*P* = 0.038). The prevalence of HBsAg positivity was not significantly different among students from the urban parts of the country (5.0%) compared to those from the rural part 2.7% (*P* = 0.273). Midwifery students were the profession most having positive HBsAg screening results (8.3%) followed by medicine (4.8%), medical laboratory (4.3%) and nursing students (3.3%). The difference was not statistically significant (*P* = 0.724).

The highest HBsAg prevalence of 8.5% (n = 11) was seen among the students who worked in hospitals compared with 2.1% (n = 5) those worked in primary health care centers, and the difference was significant (*P* = 0.012). Among the 2^nd^ year students 3.4% (n = 2) were positive to HBsAg compared with 4.6% (n = 15) among 3^rd^ year students, but the difference was not significant (*P* = 0.761). A significantly higher proportion of HBsAg positive cases were observed among students who reported history of needlestick injury than those who did not report history of needlestick injury: 6.4% vs. 2.0% (*P* = 0.024). The relation between knowledge level (good versus poor knowledge) and seroprevalence of HBV was not statistically significant (*P* = 0.077), as opposed to practice towards hepatitis B and C: prevalence of HBsAg among students experiencing poor practice was 11.0% compared to 2.5% for students experiencing good practice (*P* = 0.001).

Using multiple logistic regression analysis, the age of students and history of needlestick injury emerged as an independent predictor for HBV infection. Students in the older age group were more likely to get seropositive for HBsAg (Adjusted OR = 15.72, 95% CI 1.57–157.30) as compared to students of a younger age group. Students who experienced a needlestick injury during their clinical practice were also more likely to get seropositive for HBsAg (Adjusted OR = 3.43, 95% CI 1.10–10.73) compared to those who did not experience.

## Discussion

This study showed an overall seroprevalence rate of 4.2% for HBsAg and 0.7% for anti-HCV among medicine and health science students of Wollo University, Northeast Ethiopia. The high prevalence rate of HBV is comparable to the prevalence rate of 7.3% reported among health care workers of Southern Oromia in Ethiopia [19], and 3.2% reported among medical students of Lagos State University, Nigeria [20]. The seroprevalence rate of HBV and HCV in our study was higher than the prevalence rate of 1.7% and 0.6% for HBsAg and none for anti-HCV reported among Najran University health students of Saudi Arabia [21] and medical students of Gomal University in Pakistan [22], respectively. This may be due to most of the study participants in our study were in-service students from different health care settings. However, our prevalence rate of HBsAg was lower than 15.5% reported among medical students of Usmanu Danfodiyo University teaching hospital in Nigeria [23] and 11% reported among Makerere University Medical students of Uganda [24]. This difference may be due to the fact that more of health care workers and children are vaccinated now, as vaccine against Hepatitis B has become freely available in the country for children in May 2007[25].

The results of our study showed that the prevalence rate of HBV infection among students more than 35 years old is higher as compared to those less than 35 years of age. Students in the older age group are 15.7 times more likely to get HBV infection that may be due to they have a longer career span and they tend to ignore the universal work precaution as compared to those of a younger age group. The other possible justifications could be there may be horizontal (non-sexual contact) and vertical transmission in addition to parenteral route of transmission. On top of these they may not have access to HBV immunization in their childhood period. A statistically significant relationship between age and HBsAg positivity was also observed among students of health colleges in Saudi Arabia [26]. In our study Sero-positivity for HBsAg was 3.4 times greater among students with a history of needlestick injury. In a similar study among medical students in Uganda, unprotected exposure to patients’ body fluids and needlestick injury was found to be significantly associated with HBV infection (24). There is also evidence that after a needlestick injury in workplace situation, there is a 6–30% chance that an exposed person will be infected with HBV; indicating the need to perform regular post-exposure screening serological test.

Despite the high prevalence of Hepatitis B and C in the study participants, our results showed that overall knowledge regarding HBV and HCV infections and their mode of transmission was high (81.1%). Most respondents knew that exposure to infected blood and blood products, contaminated needles and sharps, unsafe sexual contacts, and birth to an infected mother are risk factors for hepatitis B and C infection. This finding was comparable to the knowledge levels of 86.2% reported among medicine and health sciences students of the University of Gondar hospital in Northwest Ethiopia [17], and 83.2% reported among medical students of the University of Yaounde I, Cameroon [27]. However, it was higher than the knowledge levels of 56.2% study done at Haramaya university medical and health science college in Ethiopia [18], 57.85% reported among medical students of Tanta University, Egypt [28], and 57.1% reported among medical students of seven medical colleges of Karachi, Pakistan [29].

Knowledge about prevention methods plays an important role in controlling the risk of hepatitis B and C infections during their professional training. Our study showed that higher percentage of the students had good knowledge about methods of prevention of HBV and HCV. The reason for this might be that the majority of our participants’ knowledge about hepatitis B and C was satisfactory. Our findings are consistent with the findings of other study conducted in Shiraz university of medical sciences to assess knowledge about hepatitis and prevention of the disease [30]. Another study also showed adequate level of knowledge among medical students regarding hepatitis B and C prevention measures; with 82.5% mentioning proper disposal of sharps, needles and blood, and 66.1% and 52.5% avoiding needle/sharps injury and casual sex, respectively [31].

Our study revealed that 50.0% (204/408) of the study participants had unsafe practice towards hepatitis B and C, in spite of their good knowledge regarding this life-treating disease, its mode of transmission and prevention measures. Safety practices among the study participants were poor, with only 59.8% of them properly dispose needle/ sharps; and 49.8% had history of needlestick injury. This was almost equal to the 49.9% needlestick injury reported among medical students of Karachi medical colleges/universities in Pakistan [29] and 48% among Nigerian University medical students [32]. But, it was lower than 71.1% reported among medical, dental, nursing and midwifery students of Shiraz University teaching hospital in Iran [33], and higher than 27% reported among medicine and health sciences students in Northwest Ethiopia [17] and 42.8% reported among medical school students of Nepal [34]. The high prevalence of needlestick injury in the presence of good knowledge regarding preventive measures in our study indicates that not the knowledge alone is sufficient but the implementation of the acquired knowledge that will prevent the health professional students from the risk of needlestick injuries.

Although reporting of exposure to needlestick injury is important to ensure appropriate counseling and treatment of the students, up to 46.8% of our respondents who experienced needlestick injury did not report the incidence. Failure to report needlestick injury was also recorded for 82% of students at the university teaching hospitals of Shiraz, Iran [33] and 70.3% of medical students at university teaching hospital of Karachi, Pakistan [35]. Not reporting of such injuries represents a missed opportunity for initiating post exposure prophylaxis, early detection of seroconversion and implementation of prevention strategies. Moreover, only 54.2% of our participants with a history of needlestick injury knew that a person can be infected and remain asymptomatic. Similarly, 62.9% (39 out of 62) of medical students in Cameroon had never considered the risk of HBV infection after exposure but only the risk of HIV infection [27]. Regarding post-exposure serological test, only 39.4% of the exposed students in this study had screening test result for HBV or HCV. A study conducted in Nepal medical school also found that only one-fifth of the exposed students (18 out of 90) went for a post-exposure serological test (HIV, HBV or HCV) [34].

Although this study is the first of its type, it has limitations; including that a standard enzyme linked immunosorbent assay (ELISA) method for detecting HBV and HCV markers (HBeAg, anti-HBe, IgG anti-HBc, IgM anti-HBc, viral load, and HBV DNA (for occult hepatitis)) was not included, thus possibly leading to underestimation of the actual prevalence of hepatitis B and C virus infection. The other limitations are mainly related to the fact that it was based on self reporting of the candidates and no attempt was made to revise their health records. Similarly, practices were based on the reported response of the participants and no attempt was made to directly observe their practices.

## Conclusion

The present study shows that there is high exposure and sero-prevalence of hepatitis B and C virus infection among medicine and health science students, highlighting the need for regular hepatitis B vaccination before their clinical years. The inadequate practice of the students towards occupational risk of viral hepatitis infection despite their good knowledge about the transmission and prevention measures indicates the need to protect them from health care associated infections. Another critical finding in our study is 46.8% of our respondents who experienced needlestick injury did not report the incidence. Therefore, orientation should be given regarding the transmission and prevention of HBV and HCV with particular emphasis to needlestick injury and post exposure prophylaxis before every clinical attachment.

## Supporting information

S1 File Questionnaire used collect sociodemographic characteristics and KAP items of study participants. (DOCX)

## Abbreviations

Anti-HCV: anti-hepatitis C virus
AOR: adjusted odds ratios
CI: confidence intervals
HBsAg: hepatitis B surface antigen
HBV: hepatitis B virus
HCV: hepatitis C virus
