# Supplementary material for: Hepatitis B and C: Seroprevalence, knowledge, practice and associated factors among medicine and health science students in Northeast Ethiopia
Source: PLoS One. 2018 May 15;13(5):e0196539. doi: 10.1371/journal.pone.0196539 (PMC5953438; doi:10.1371/journal.pone.0196539)
Supplement: S1 File — (DOCX) [file pone.0196539.s001.docx]

## Annex I: questionnaire used collect sociodemographic characteristics and KAP items of study participants.

Date ______________

Ser. NO_____________

**Part I. Socio-demographic characteristics and other factors (encircle for those having options and write your own on the space provided):**

1. Age: _________
2. Sex:
3. Male
4. Female
5. Marital status:
6. Single
7. Married
8. Divorced
9. Widowed
10. Residence:
11. Urban
12. Rural
13. Department:
14. Environmental Health
15. Medical Lab
16. Midwifery
17. Nursing
18. Pharmacy
19. Public health
20. Others
21. Year of study
22. 1^st^
23. 2^nd^
24. 3^rd^
25. 4^th^
26. 5th

**Part II. Questions on knowledge on background, disease, transmission, treatment, prevention and other factors (encircle for those having options and write your own the space provided):**

1. Hepatitis can be caused by virus.
2. Yes
3. No
4. Do you think Hepatitis B and C can be transmitted through
5. Blood and blood products
6. Yes
7. No
8. Needle and sharp
9. Yes
10. No
11. Sexual intercourse
12. Yes
13. No
14. Feco-oral and contaminated water
15. Yes
16. No
17. Nosocomial infection
18. Yes
19. No
20. Mother to fetus
21. Yes
22. No
23. Close personal contact like kissing/ talking
24. Yes
25. No
26. Do you think you are safe from acquiring any infection via patients?
27. Yes
28. No
29. Do you know that all HCWs can acquire hepatitis B and C infection owing to their professional contact with their patients?
30. Yes
31. No
32. Do you think that all HCWs should be routinely tested for Hepatitis B and C
33. Yes
34. No
35. Do you believe in restricting hepatitis B and C-positive health care workers to low-risk procedures?
36. Yes
37. Yes
38. Do you know the presence of vaccine for HBV?
39. Yes
40. No
41. Do you know the presence of vaccine for HCV?
42. Yes
43. No
44. Do you think the following methods used to prevent Hepatitis B and C infection?
45. Vaccination
46. Yes
47. No
48. Proper disposal of sharps, needle and blood
49. Yes
50. No
51. Avoiding needle/sharps injury
52. Yes
53. No
54. Avoid causal sex/multi sexual partnership
55. Yes
56. No
57. Avoid drinking contaminated water
58. Yes
59. No
60. Avoid eating not well cooked food
61. Yes
62. No
63. There is pharmaceutical treatment for hepatitis B
64. Yes
65. No
66. Hepatitis B can cause liver cancer
67. Yes
68. No
69. Do you always use glove while handling different body fluids
    1. Yes
    2. No
70. Do you always properly dispose needle/ sharps
    1. Yes
    2. No
71. Do you have had a history of needlestick injury?
    1. Yes
    2. No
72. If yes have you reported it?
    1. Yes
    2. No
73. Have you ever screened for hepatitis B or C?
    1. Yes
    2. No
74. A person can be infected with hepatitis B and C and not have any symptom of the disease
75. Yes
76. No

Name of interviewer __________________________

Signature _________________
